# Correction: Liu et al. Emergent Peptides of the Antifibrotic Arsenal: Taking Aim at Myofibroblast Promoting Pathways. *Biomolecules* 2023, *13*, 1179

**DOI:** 10.3390/biom14040407

**Published:** 2024-03-27

**Authors:** Zhen Liu, Xinyan Zhang, Yanrong Wang, Yifan Tai, Xiaolin Yao, Adam C. Midgley

**Affiliations:** 1 State Key Laboratory of Medicinal Chemical Biology, Key Laboratory of Bioactive Materials for the Ministry of Education, College of Life Sciences, Nankai University, Tianjin 300071, China; 2 School of Food and Biological Engineering, Shaanxi University of Science and Technology, Xi’an 710021, China

There was an error in the original publication [1]. One of the reviewed peptides, THR-184, completed phase 2 clinical trials in 2017, but for a different disease treatment. A correction has been made to Section 5. Discussion and Future Perspectives, paragraph 4:

“Most of the peptides reviewed here are in the preclinical stage of development and have yet to enter clinical trials. THR-184 and ANG-3777 (an HGF peptide mimetic previously known as BB3) have both completed phase 2 clinical trials (ClinicalTrials.gov ID: NCT01830920; NCT02771509) as perioperative treatments in cardiovascular-surgery-associated acute kidney injury (CVS-AKI), which is a type of ischemic injury that may progress to chronic kidney disease and renal fibrosis [59,60].”

With this change, two more references were added: [59] and [60] (see [2,3]), and the original [59] and [60] have been modified to [61] and [62].

The authors state that the scientific conclusions are unaffected. This correction was approved by the Academic Editor. The original publication has also been updated. 

## References

[1] Liu Z., Zhang X., Wang Y., Tai Y., Yao X., Midgley A.C. (2023). Emergent Peptides of the Antifibrotic Arsenal: Taking Aim at Myofibroblast Promoting Pathways. Biomolecules.

[2] Carlson W.D., Keck P.C., Bosukonda D., Carlson F.R. (2022). A Process for the Design and Development of Novel Bone Morphogenetic Protein-7 (BMP-7) Mimetics with an Example: THR-184. Front. Pharmacol..

[3] Ayad S., Neylan J.F., Mayne T.J., Gouveia D., Swaminathan M. (2022). Hepatocyte Growth Factor Mimetic ANG-3777 for Cardiac Surgery–Associated Acute Kidney Injury. Kidney Int. Rep..

